# Supplementary material for: DNA Repair Inhibitors Potentiate Fractionated Radiotherapy More Than Single-Dose Radiotherapy in Breast Cancer Cells
Source: Cancers (Basel). 2022 Aug 4;14(15):3794. doi: 10.3390/cancers14153794 (PMC9367425; doi:10.3390/cancers14153794)
Supplement: Supplementary file 1 [file cancers-14-03794-s001.zip › Supplementary Figure S1.pdf]

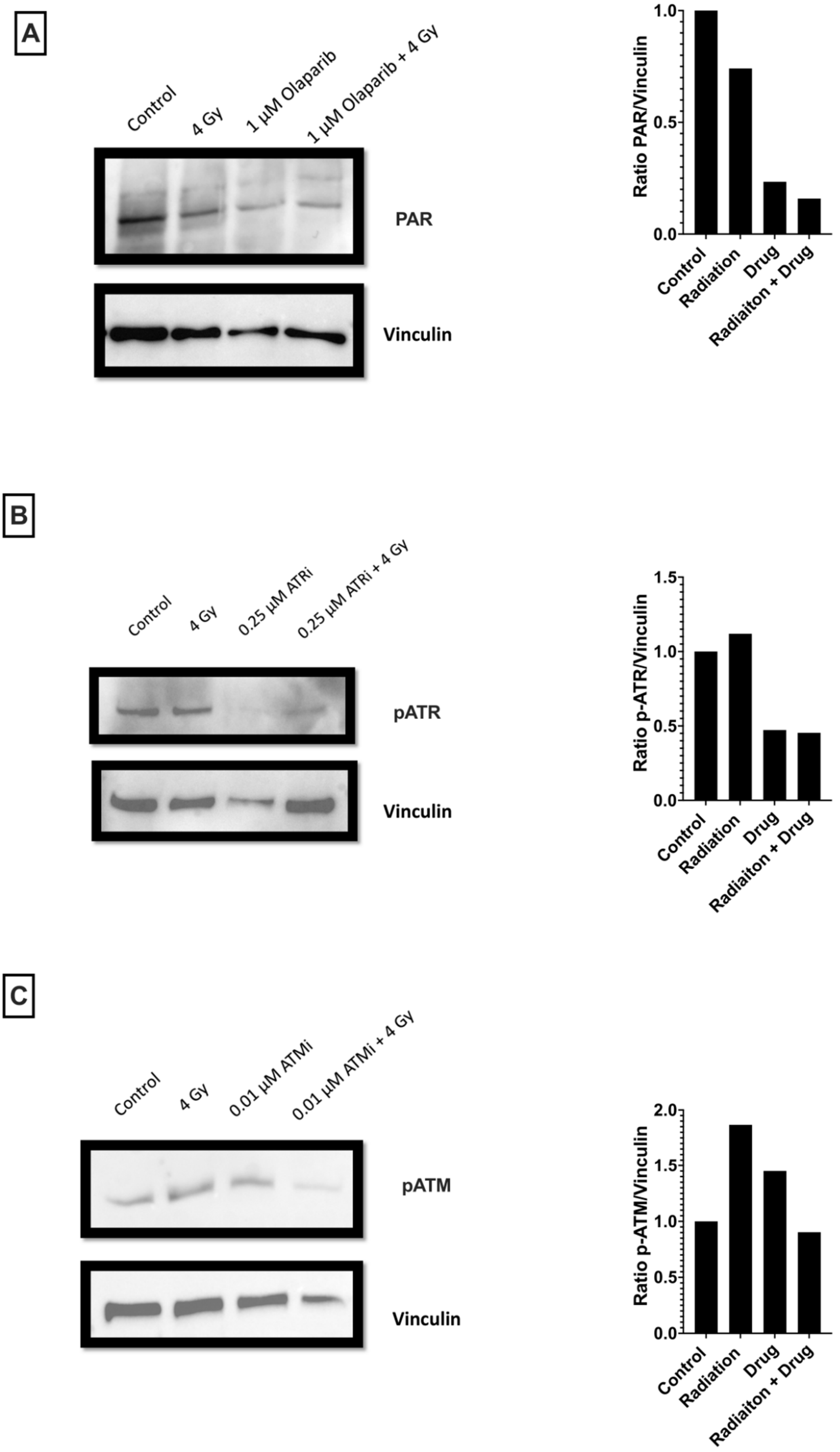
A
Control
4 Gy
1 µM Olaparib
1 µM Olaparib + 4 Gy
PAR
Vinculin
Ratio PAR/Vinculin
1.0
0.5
0.0
Control
Radiation
Drug
Radiaiton + Drug
B
Control
4 Gy
0.25 µM ATRi
0.25 µM ATRi + 4 Gy
pATR
Vinculin
Ratio p-ATR/Vinculin
1.5
1.0
0.5
0.0
Control
Radiation
Drug
Radiaiton + Drug
C
Control
4 Gy
0.01 µM ATMi
0.01 µM ATMi + 4 Gy
pATM
Vinculin
Ratio p-ATM/Vinculin
2.0
1.5
1.0
0.5
0.0
Control
Radiation
Drug
Radiaiton + Drug

Supplementary Figure S1. Effect of AZD0156, AZD6738 and olaparib on target proteins of respective DNA repair pathways in MDA-MB-231 cells. (**A**) Olaparib directly affects the protein levels of PAR. (**B**) AZD6738, an ATR inhibitor, directly affects phosphorylation of ATR; while (**C**) AZD0156, an ATM inhibitor, directly affects phosphorylation of ATM. Phosphorylation or protein levels were evaluated for control cell populations only treated with DMSO, cells only treated with 4 Gy of radiation, cells only treated with DDR inhibitors, or cells treated with a combination of both. Levels of Vinculin were used as a loading control, and quantification was performed with ImageJ. Protein ratios were normalized to the untreated control cells.
